# IM-MetaLAB: the first digital laboratory for teaching the fundamental concepts of instrumentation and measurement in metaverse

**DOI:** 10.1038/s41598-025-29553-7

**Published:** 2025-11-26

**Authors:** Francesco Bonavolontà, Antonio Monaco, Enzo Caputo, Annalisa Liccardo

**Affiliations:** 1 Department of Electrical Engineering and Information Technology, University of Naples Federico II, Naples, 80125 Italy; 2 GAV Projects s.r.l., S. Prisco (Caserta), 81054 Italy; 3 Meditech Competence Center, Naples, 80146 Italy

**Keywords:** Metaverse laboratory, Digital twin, Instrumentation training, Virtual reality education, IoT in education, Engineering, Mathematics and computing

## Abstract

This paper presents the Instrumentation and Measurements Metaverse Laboratory (IM-MetaLAB), an immersive virtual laboratory developed within the metaverse to support hands-on training in instrumentation and measurement. Unlike previous Augmented Reality (AR)/Virtual Reality (VR)-based solutions or simulated environments, IM-MetaLAB integrates real laboratory instruments through digital twins that communicate in real time with their physical counterparts using Standard Commands for Programmable Instruments (SCPI) commands over Message Queuing Telemetry Transport (MQTT). The platform supports synchronous and asynchronous learning modes, promotes collaborative activities among students, and replicates real-world laboratory procedures in a safe and accessible 3D environment. A representative case study, focused on root means square (RMS) voltage measurement, demonstrates the system’s capabilities in executing full experimental workflows. Comparative analysis with traditional, online, and remote lab-based teaching methods was conducted using key performance indicators such as student engagement, completion rate, and examination scores. The results indicate that IM-MetaLAB significantly improves learning outcomes and practical proficiency. Ongoing work addresses scalability and cost-reduction through the implementation of a custom hardware interface.

## Introduction

The Coronavirus Disease 2019 (COVID-19) pandemic has profoundly transformed the landscape of higher education, prompting universities to rapidly migrate courses and laboratories online. This shift affected over 220 million students and 25,000 institutions worldwide^1^, compelling traditional academia to adopt digital teaching strategies previously explored only by a few pioneering institutions.

While online education proved effective under certain conditions—especially due to its flexibility in overcoming geographical and temporal constraints—it also revealed critical limitations, particularly in Science, Technology, Engineering, and Mathematics (STEM) fields. The challenge lies in replicating hands-on laboratory experiences, which are fundamental for teaching instrumentation and measurement^2,3^. Various solutions have emerged to address this, including remote laboratories^4–8^, which allow students to control real instruments over the internet, and widely adopted systems such as Massachusetts Institute of Technology (MIT) iLab^9^ and the Virtual Instruments Systems In Reality (VISIR) Lab^10^.

Despite their strengths—primarily the use of real equipment—remote labs often rely on simple user interfaces, which limit the development of operational familiarity and reduce immersion^11–13^. Augmented Reality labs have been proposed to overcome this, offering interactive 3D replicas of physical instruments^14^. While AR improves realism, it still lacks support for collaborative work and circuit assembly, and offers limited interaction with small components like connectors.

The evolution of Virtual Reality technologies now enables the creation of immersive 3D environments that simulate physical classrooms and labs with high fidelity. This concept, increasingly referred to as the *Metaverse*, allows students and instructors to interact via avatars in persistent, collaborative virtual spaces. Academic applications include enhanced engagement, spatial reasoning, and real-time manipulation of digital twins of laboratory instruments, particularly in engineering and healthcare education.

Within this context, the IM-MetaLAB is presented hereinafter: a fully immersive, metaverse-based remote laboratory that integrates 3D digital twins of physical instruments synchronized in real-time via Internet of Things (IoT) protocols. IM-MetaLAB enables both synchronous and asynchronous learning, collaborative exercises, and a realistic replica of a typical measurement setup for lab activities, thus overcoming many of the technical limitations of earlier solutions.

The remainder of the paper is organized as follows. Section "The role of the Metaverse in didactics and vocational training: state of the art" reviews the role of the Metaverse in education. Section "The IM-MetaLAB" describes the IM-MetaLAB and its functionalities. Section "The framework of IM-MetaLAB" details the supporting technological framework. In Sect. "Case study: measurement of RMS voltage through the multimeter", a case study on RMS voltage measurement is presented. Section "Analysis of KPIs" analyzes the key performance indicators (KPIs), and Section “Summary” concludes the paper.

## The role of the Metaverse in didactics and vocational training: state of the art

The Metaverse is emerging as a promising framework for improving higher education by enabling immersive, persistent, and collaborative learning environments^15,16^. Through avatar-based interactions in virtual 3D spaces, students can engage in lectures, laboratories, and collaborative projects, potentially overcoming the spatial, temporal, and logistical constraints of traditional or remote learning setups.

Several universities have developed metaverse-based platforms tailored to specific disciplines. For instance, Stanford’s Virtual Physics Lab enables students to explore complex physical phenomena such as quantum mechanics or cosmic-scale simulations, which would be impossible in real-world labs^17^. Similarly, Harvard’s Virtual Chemistry Lab allows experimentation with simulated substances and reactions in a safe digital setting^18^, while Eidgenössische Technische Hochschule (ETH) Zurich’s Robotics Lab supports algorithm development in simulated environments^19^.

However, in all these cases, the educational experience remains confined to a virtual-only context, based on simulated models with no direct coupling to physical laboratory equipment. Although they are valuable for conceptual exploration and safety, these systems lack real-time interaction with actual instruments (or even don’t require for it), thus limiting the development of operational competencies and practical familiarity.

Some initiatives, like those at the University of Tokyo^20^ or National University of Singapore (NUS)^21^, explore highly realistic and collaborative virtual environments for environmental sciences or healthcare training. Yet, their implementations generally rely on predefined simulation scenarios, and do not feature the bidirectional data flow or interactive digital twins that characterize a true cyber-physical lab environment.

Unlike these approaches, the proposed IM-MetaLAB combines the immersive advantages of the Metaverse with direct, real-time interaction with physical laboratory instruments. Students interact with photorealistic digital twins of actual devices that are continuously synchronized via IoT protocols (e.g., MQTT). This ensures that any virtual action—such as turning a knob or taking a measurement—corresponds to an actual command executed on the physical device. Consequently, IM-MetaLAB bridges the gap between immersive simulation and hands-on realism, offering an authentic and measurable educational experience.

Furthermore, IM-MetaLAB supports both synchronous and asynchronous learning, with built-in tools for data visualization, collaboration, and even informal social interaction. It also allows for automated activity tracking and personalized feedback—features often missing in prior Metaverse applications.

Finally, in vocational and industrial training, several projects have highlighted the potential of virtual environments to simulate complex or hazardous procedures^22,23^. Yet, most solutions rely on static scenarios or pre-recorded behavior models, and lack the flexibility, instrument fidelity, and real-world integration needed for comprehensive instrumentation training.

Unlike systems based on preconfigured simulations or remote-only access, IM-MetaLAB enables immersive interaction with actual hardware through real-time bidirectional synchronization, which is not present in prior Metaverse-based education systems. Morevoer, IM-MetaLAB provides a fully interactive and scalable platform, where real instruments and digital counterparts operate in parallel. This hybrid approach ensures skill transferability, enhances student autonomy, and offers an unprecedented level of realism and inclusivity in STEM education.

## The IM-MetaLAB

### Spaces appearance and digital twins of the instruments

The user experience in Metaverse environments relies on the combined effects of immersion and presence, which enable realistic and engaging interaction with virtual worlds^24^. Immersion depends on technical fidelity, including accurate rendering, stable frame rates, and full-motion head tracking, all of which contribute to the illusion of being surrounded by a coherent virtual space. Presence, on the other hand, is established through avatars and real-time embodied interaction: users select and control their digital identity via motion-tracked controllers, allowing them to manipulate objects and navigate the environment. This sense of being virtually present is further amplified by multi-presence, where co-located avatars foster synchronous and spatial collaboration—an essential component for meaningful educational and communicative experiences within the Metaverse.

Within this immersive and social framework, the quality of the virtual scene plays a critical role in shaping the learning experience. Traditional virtual learning environments often replicate real classrooms using rigid geometries and familiar furnishings such as desks, chairs, and blackboards^25,26^. While these representations offer familiarity, they do not fully exploit the expressive freedom of VR. The IM-MetaLAB platform adopts a more innovative approach to scene design, using the affordances of immersive 3D space to create a more engaging and fluid environment.

**Fig. 1:**
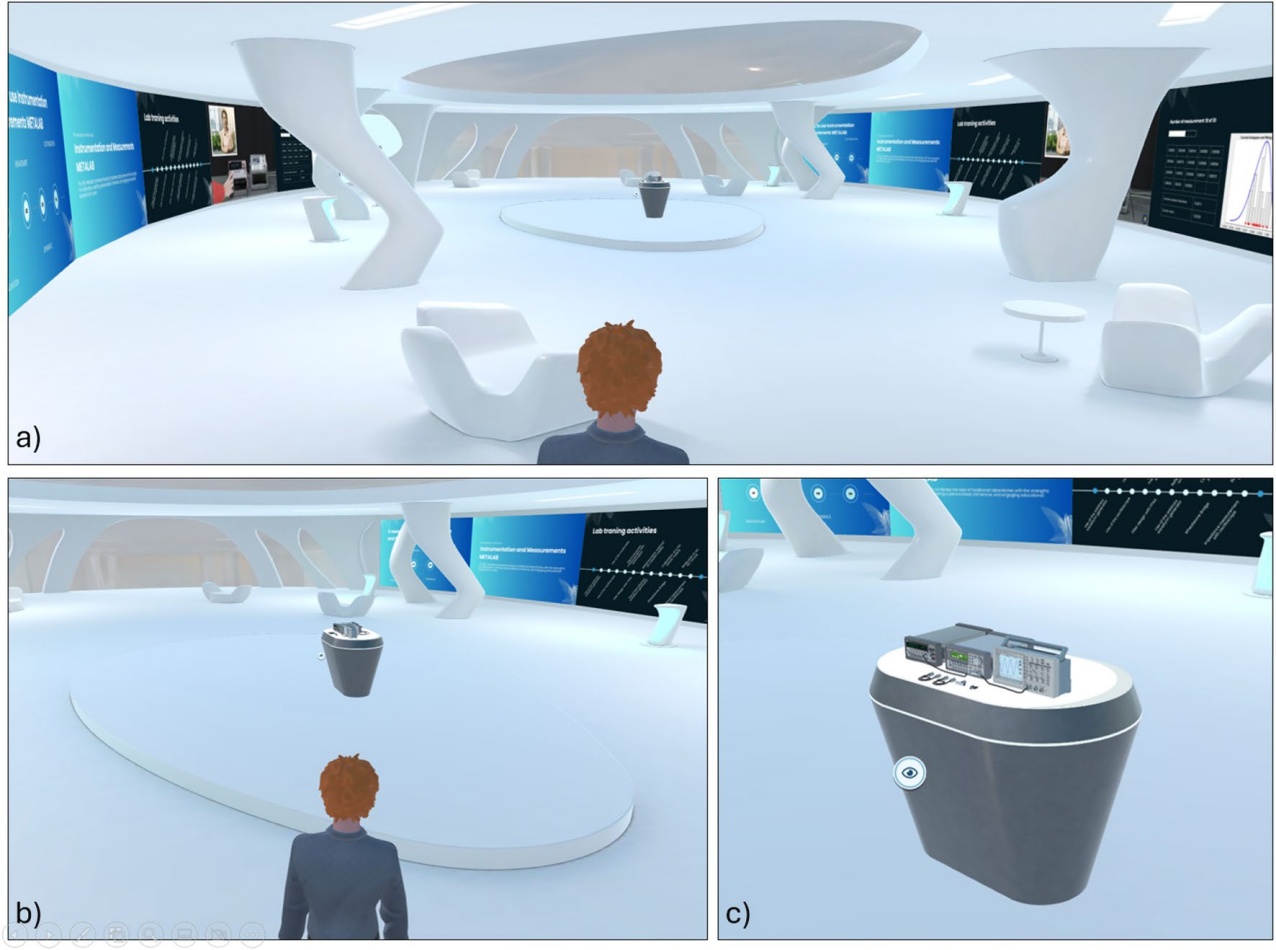
Learning scene of the IM-MetaLAB. The center of the scene is characterized by the measurement setup, while interactive walls with videos, slideshow and interactive dashboards improve the immersiveness for the student.

The virtual lab is modeled as an elliptical classroom that replaces rigid corners with curved geometries, establishing a sense of spatial continuity and enhancing the centrality of the interaction space (Fig. 1a). This design favors inclusivity, visibility, and a shared focus, overcoming the spatial limitations of traditional layouts. The room is rendered with light tones, minimalist surfaces, and soft lighting, which supports both functional clarity and psychological comfort. It should be noted that the considered dimension of the classroom mainly prevent the effects of motion sickness usually associated with the VR headset. Once chosen the position in the classroom (either for listening or operating with the measurement setup), the user, in fact, no longer needs to move, thus avoiding the occurrence of this harmful problem.

**Fig. 2:**
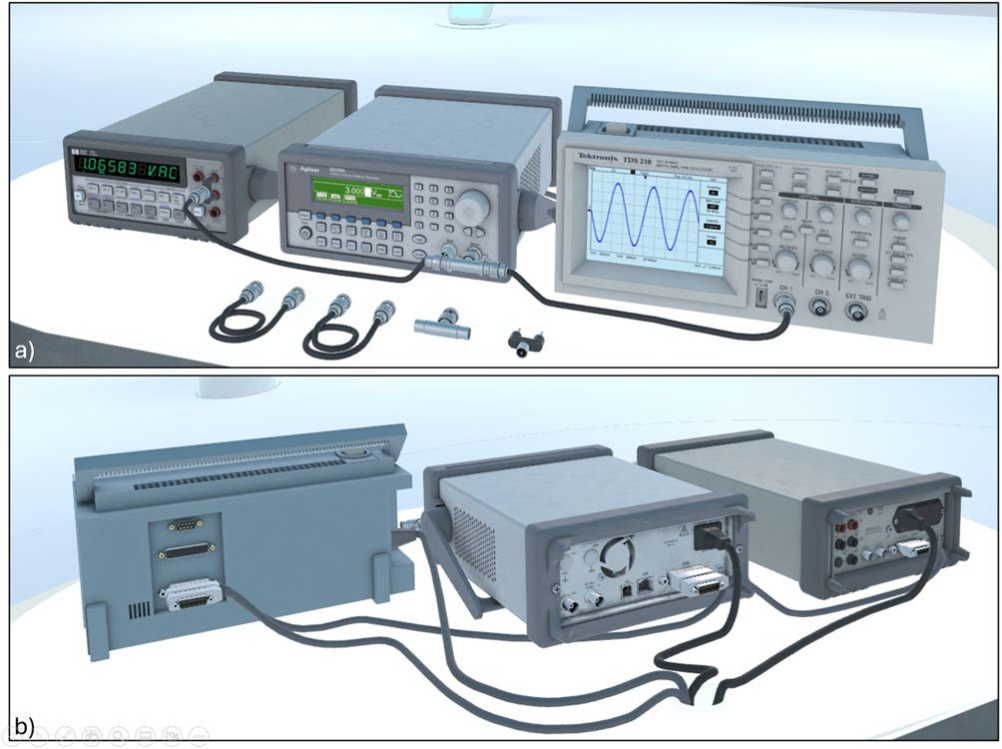
The measurement setup consists of an arbitrary waveform generator, a digital multimeter and a digital storage oscilloscope, the fundamental instruments for most measurements and instrumentation courses. Instruments representation (**a**) Front View (**b**) Back View.

At the center of the space is a circular stage slightly elevated from the floor, hosting a table on which interactive 3D replicas of the laboratory instruments are placed (Fig. 1b,c). The central positioning of this table serves as a visual and functional anchor for the session: it guarantees equal visibility for all participants and encourages active exploration and discussion of the virtual devices.

Stemming from prior work in augmented reality labs^14^, the authors have developed highly realistic 3D models of measurement instruments, now further enhanced for immersive rendering in the Metaverse^27^. It should be noted that the these digital twins are not just static and aesthetic model of the measurement setup: they are synchronized in real-time with their physical counterparts via IoT communication protocols.

To achieve high fidelity, each model replicates the instrument from all angles (Fig. 2) with fine-grained details such as buttons, knobs, connectors, and cooling systems, following rendering practices from 3D visualization standards^28^.

Going beyond visual realism, the environment supports direct interaction with cables and connectors, as Bayonet Neill–Concelman (BNC), which users can grab and connect using hand-tracked VR controllers. This functionality enables students to build full measurement circuits, closely mimicking physical lab operations and promoting critical hands-on skills that are usually inaccessible in traditional remote or AR-based solutions. Wrong operations are highlighted to the students and recorded in their connection and activity logs.

### Additional learning functionalities

**Fig. 3:**
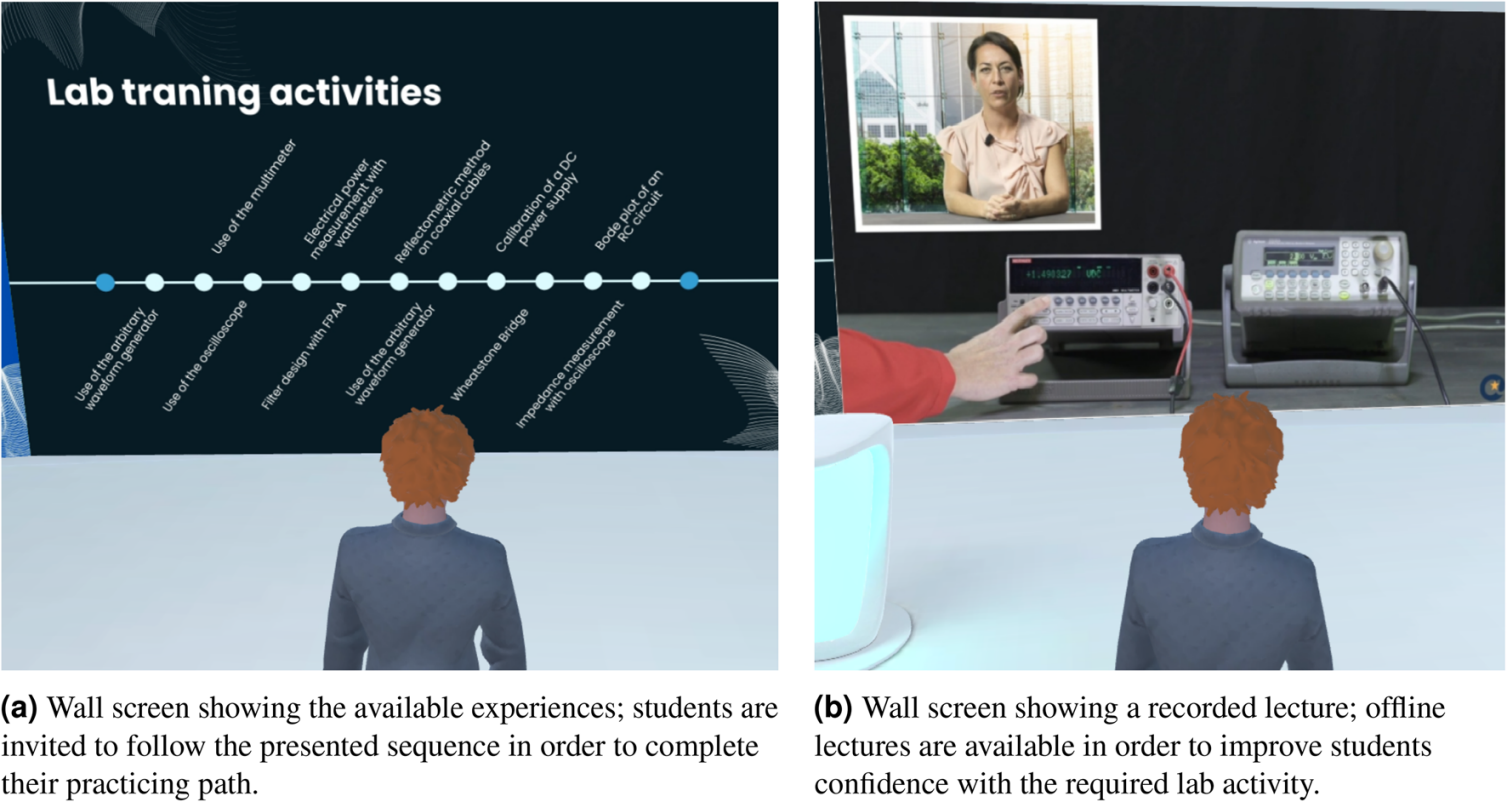
Examples of wall screens in the IM-MetaLAB capable of enriching the didactic experience.

The IM-MetaLAB includes wall screens strategically placed in the environment to support different didactic goals (Fig. 3). These displays can present real-time data, pre-recorded lectures, multimedia content, dashboards, and 3D animations, thus enriching the learning process.

These screens also function as shared dashboards where groups can visualize data from instruments in real-time, perform statistical analysis, or receive automated feedback during exercises. This multi-modal access enhances student engagement and enables differentiated instruction.

**Fig. 4:**
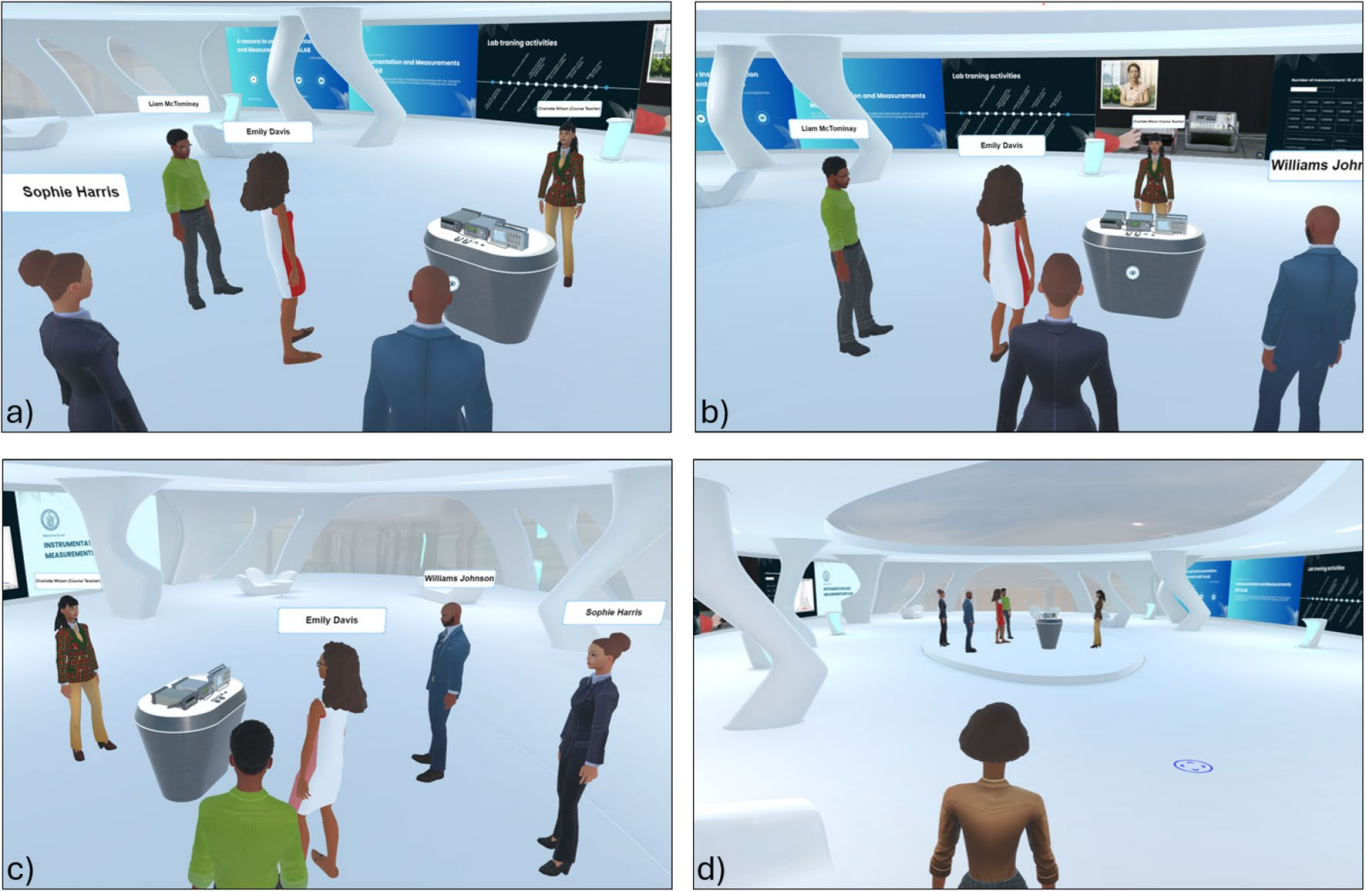
Different viewpoints for the synchronous learning activity; the lecturer can use synchronous activity to show to the students the operating steps of the lab experience as well as check their operations during a successive assessment stage or personal experiment execution. The availability of audio and microphone connections allows the lecturer to interact with all the students attending the class.

The IM-MetaLAB supports both synchronous sessions—where the entire class gathers with the instructor in real-time (Fig. 4)—and asynchronous activities. In synchronous mode, students can interact verbally or via gestures, watch live demonstrations, and collaborate on the same setup.

**Fig. 5:**
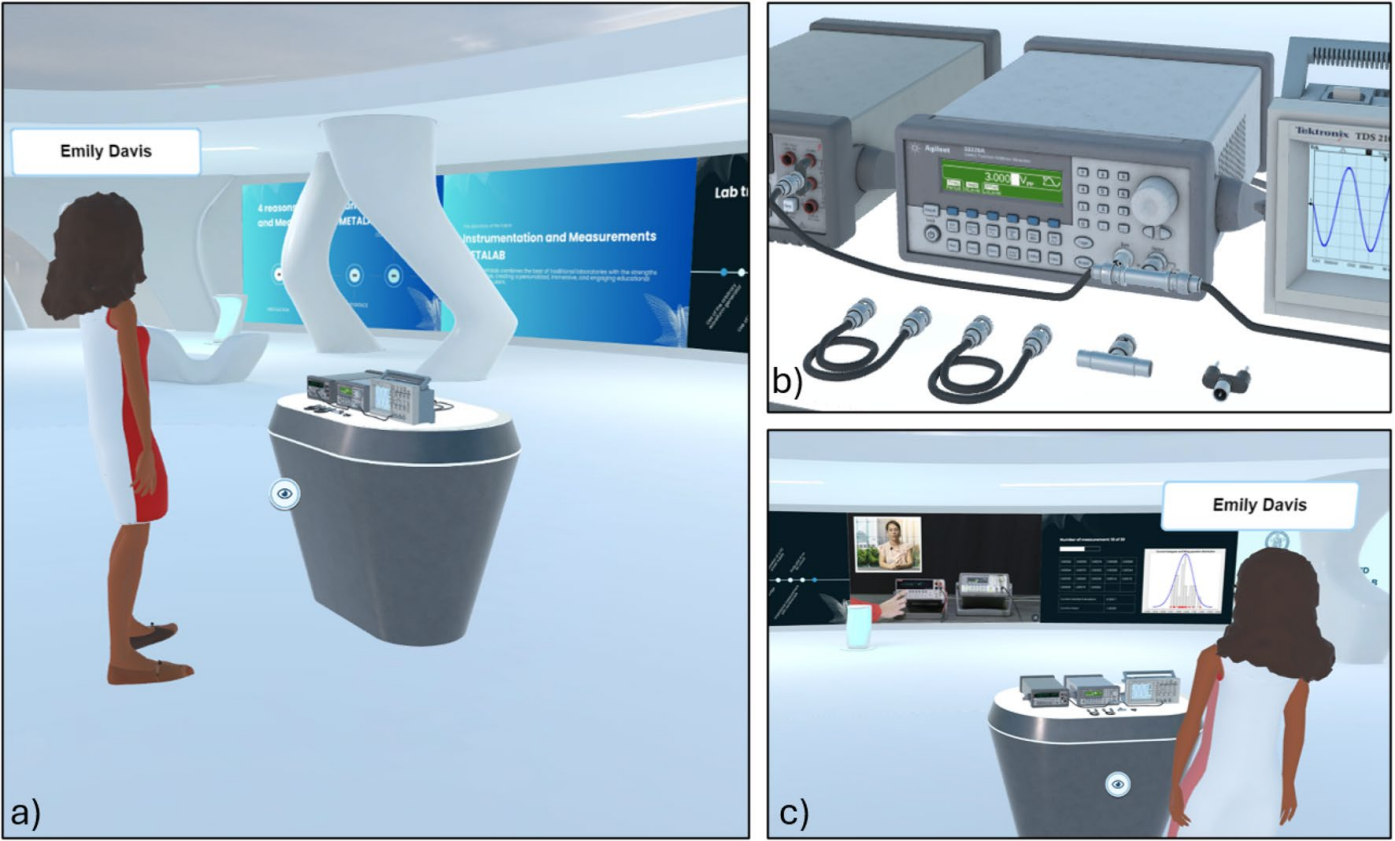
The 24 h availability of the IM-MetaLAB allows students to access the lab facilities also for asynchronous learning activity (here shown by different viewpoints); they can use the measurement setup to repeat the required experiments to improve their capability or try to understand how different instrument configurations can affected the measurement quality. Also in this case, the connection log allows teacher to assess students operations, if necessary.

Outside scheduled sessions, students can independently access the lab, repeat experiments, and review supporting materials (Fig. 5). All resources—cables, instruments, manuals, and videos—remain accessible, enabling self-paced learning and skill consolidation.

**Fig. 6:**
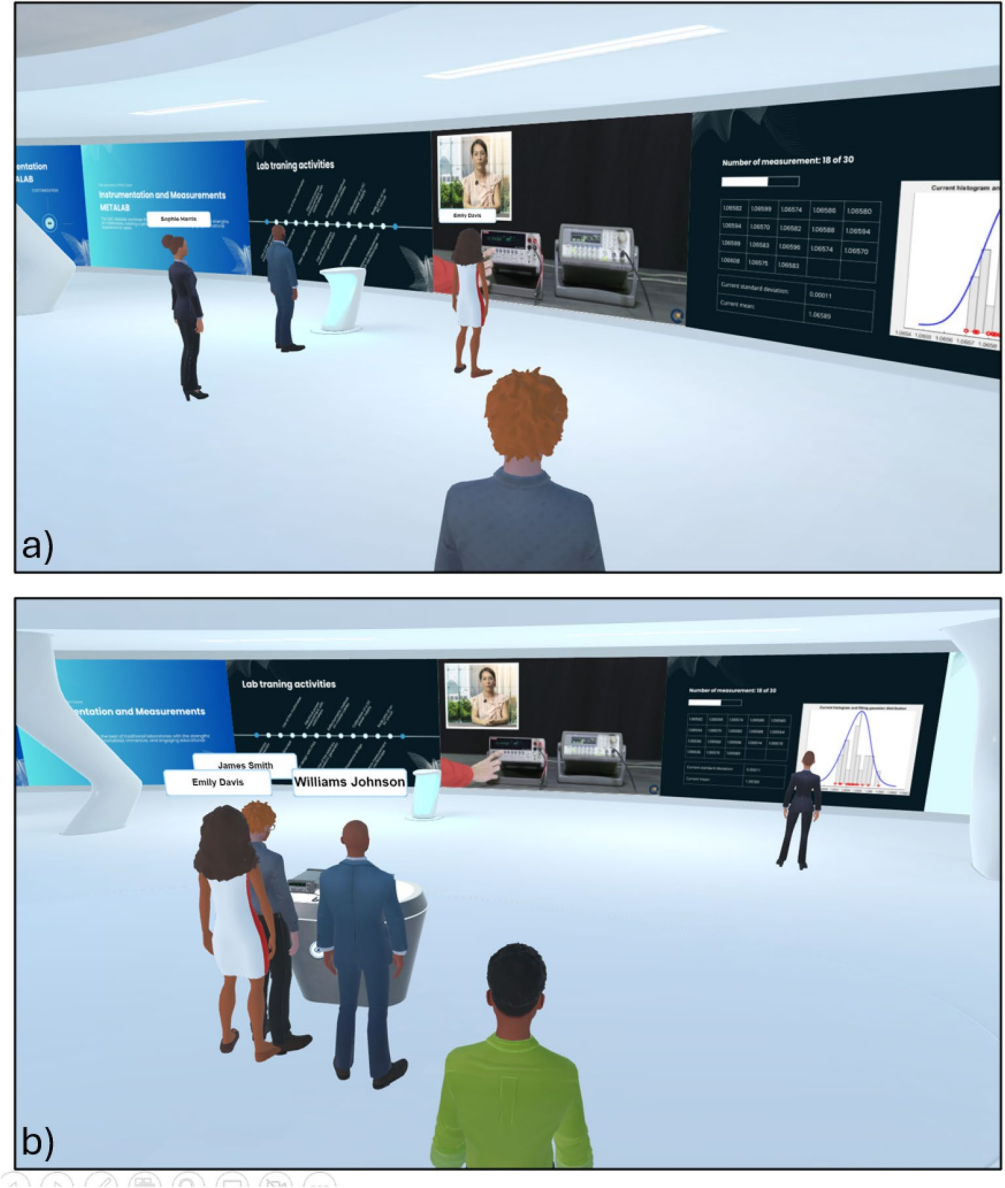
Asynchronous group activities (different viewpoints) allow students to share their knowledge and opinions outside the traditional class period and cooperate to accomplish lab experiences.

Students may also meet in the Metaverse to conduct collaborative exercises, emulating real-life teamwork (Fig. 6).

**Fig. 7:**
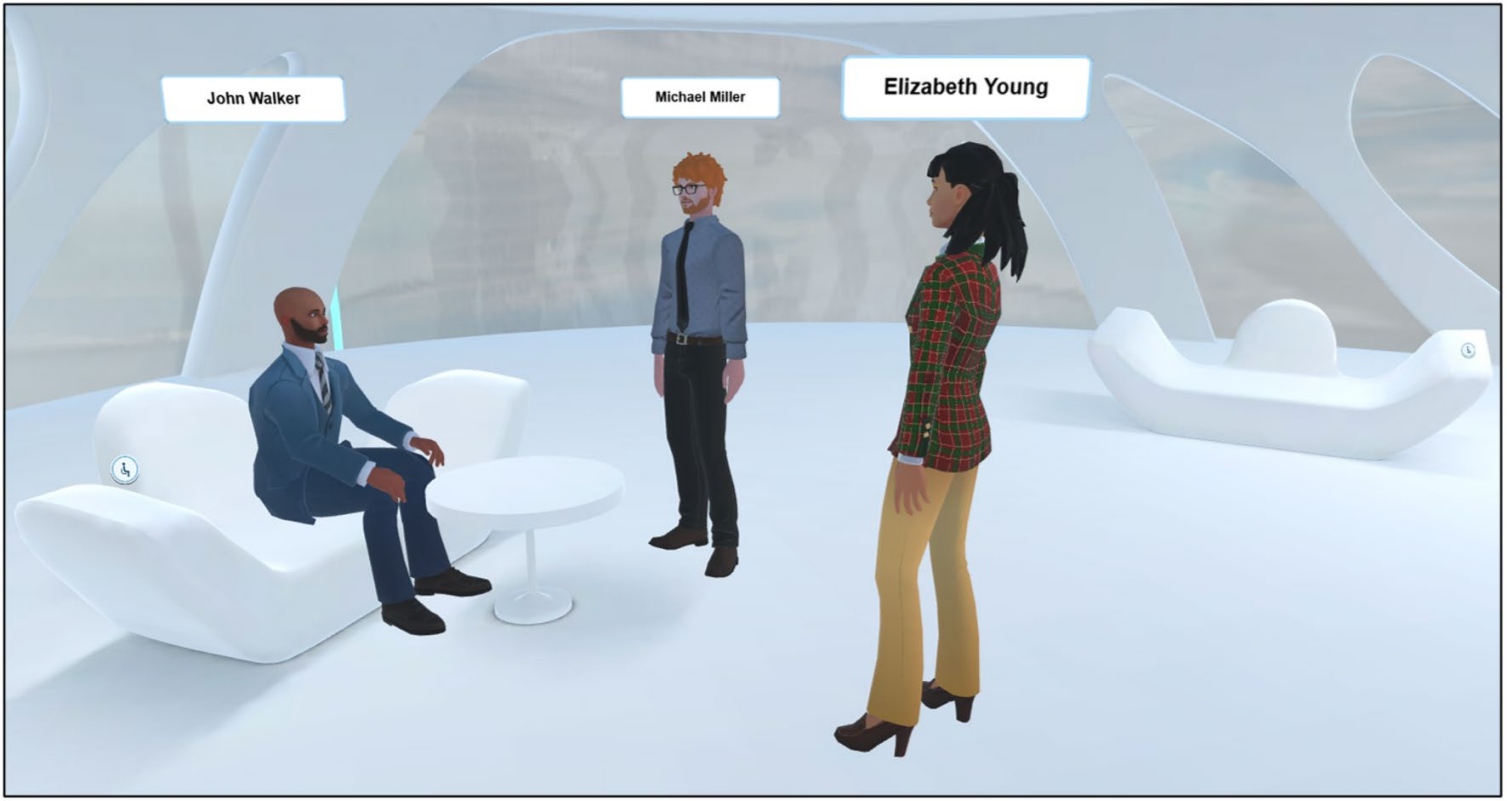
As for other metaverse applications, students can meet during an informal meeting; the IM-MetaLAB can, in fact, be exploited to have social interactions not directly related with the didactic experience.

In addition to formal learning spaces, IM-MetaLAB includes informal zones for unstructured interactions (Fig. 7). These social areas—equipped with virtual tables and seating—allow students to engage in non-academic discussions, reinforcing the sense of presence and community that often lacks in remote education environments.

## The framework of IM-MetaLAB

The Metaverse Laboratory does not “reside” in a physical location, but “lives on the internet,” utilizing a network infrastructure composed of high-performance hardware components. This includes servers equipped with: 1) powerful Graphics Processing Units (GPUs) capable of rendering realistic environments in real-time; 2) sufficient storage space to ensure that multiple users can simultaneously access the Metaverse Laboratory (Multipresence), sharing the same virtual space so that everyone can see and experience the same events and interactions in real-time.

**Fig. 8:**
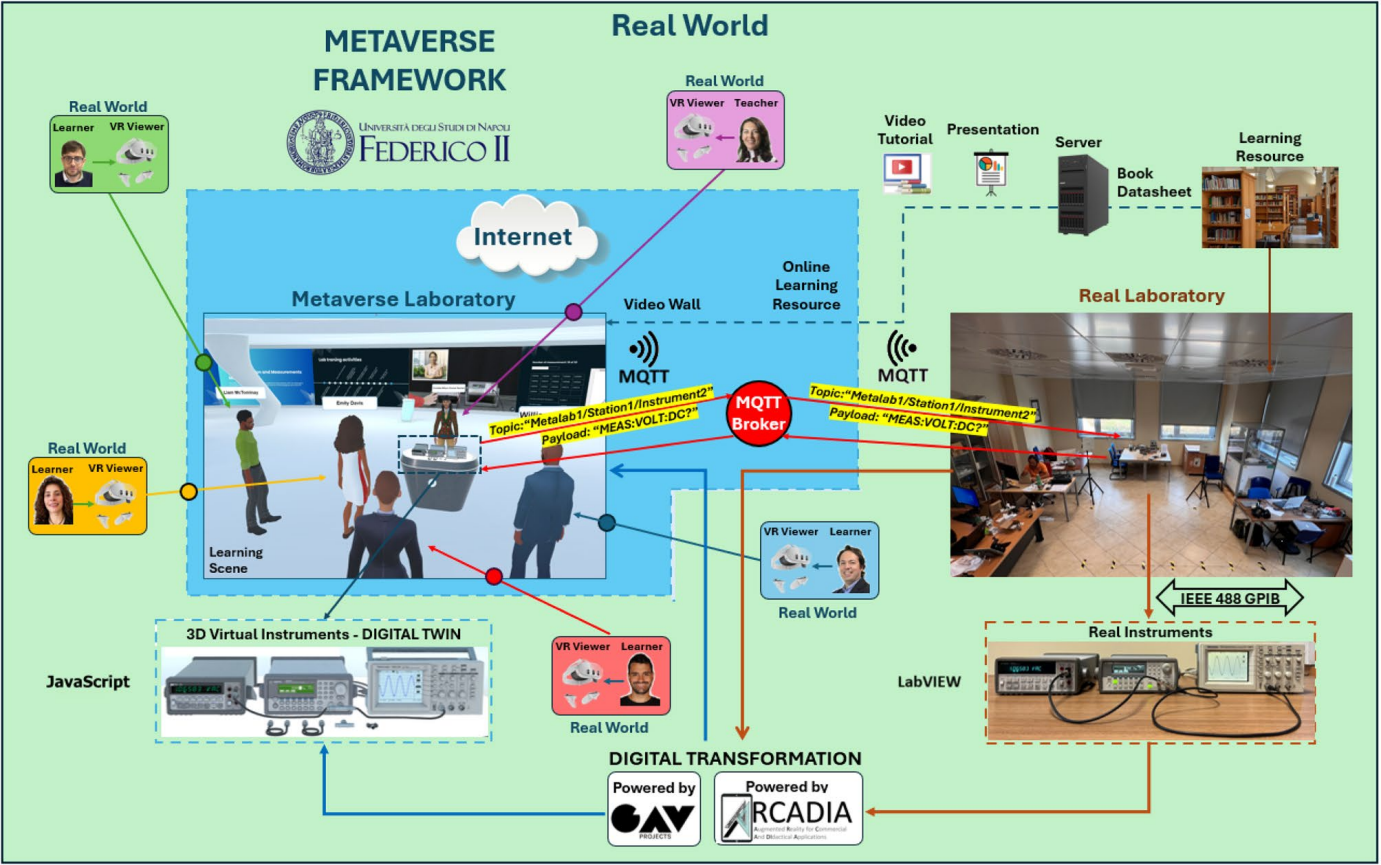
Framework of the IM-MetaLAB; javascript-powered replicas of the instruments are controlled by users and connected to the actual measurement setup thank to an MQTT-based messaging protocol. A PC running a LabVIEW server at lab-side allows to convert MQTT messages into the corresponding commands/queries the instruments can understand and undergo to provide the required measurement result.

For this specific application, the hosting hardware consists of a high-performance workstation equipped with an AMD Ryzen Threadripper PRO 5975WX processor running at 4.5 GHz, four Nvidia Quadro RTX A6000 GPUs with 48 GB of GDDR6 memory and a 384-bit interface, and a 7.68TB SSD data storage unit. The framework underlying the IM-MetaLAB Lab is illustrated in Fig. 8.

**Fig. 9:**
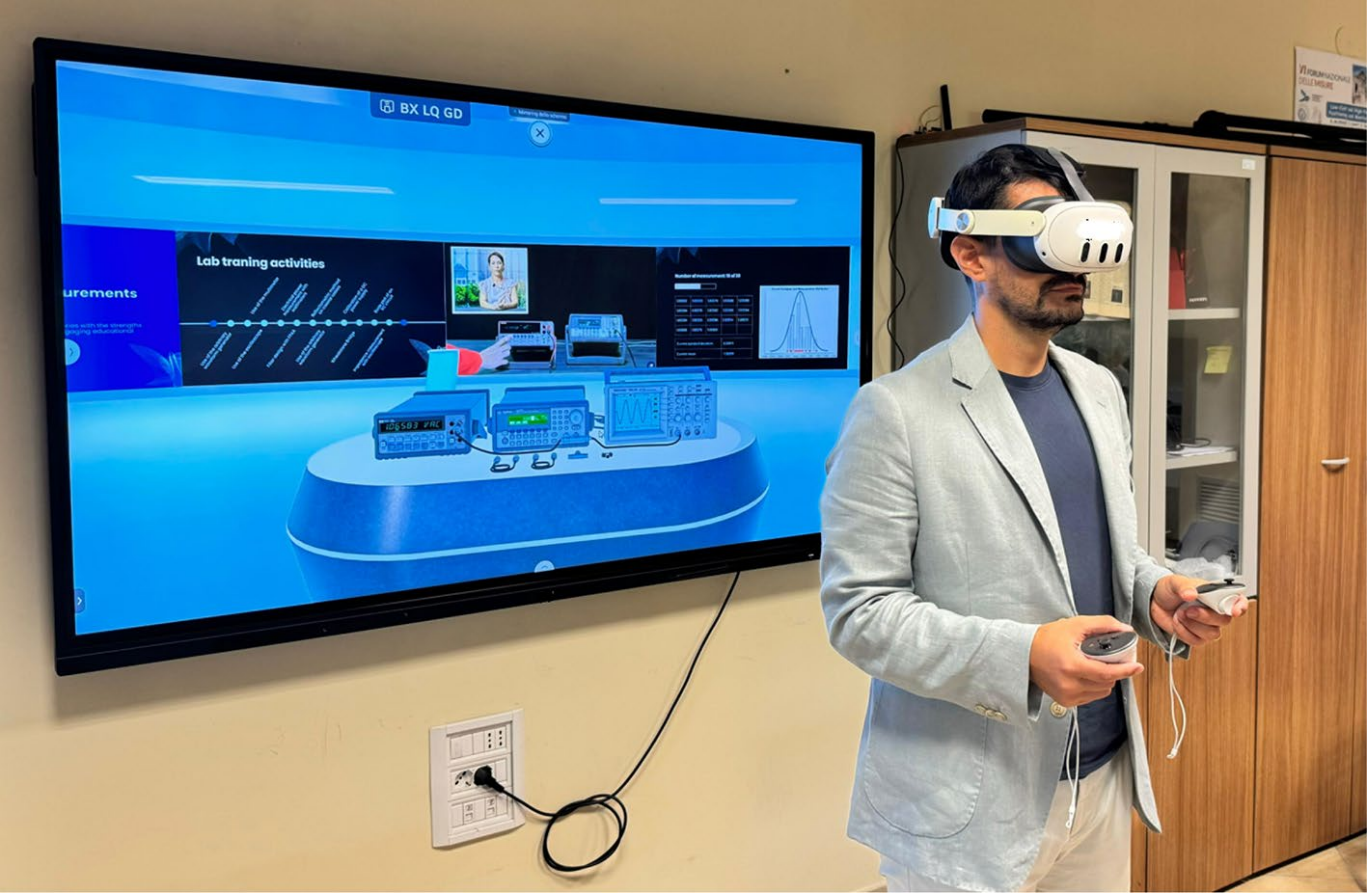
User wearing VR device for accessing the IM-MetaLAB.

**Fig. 10:**
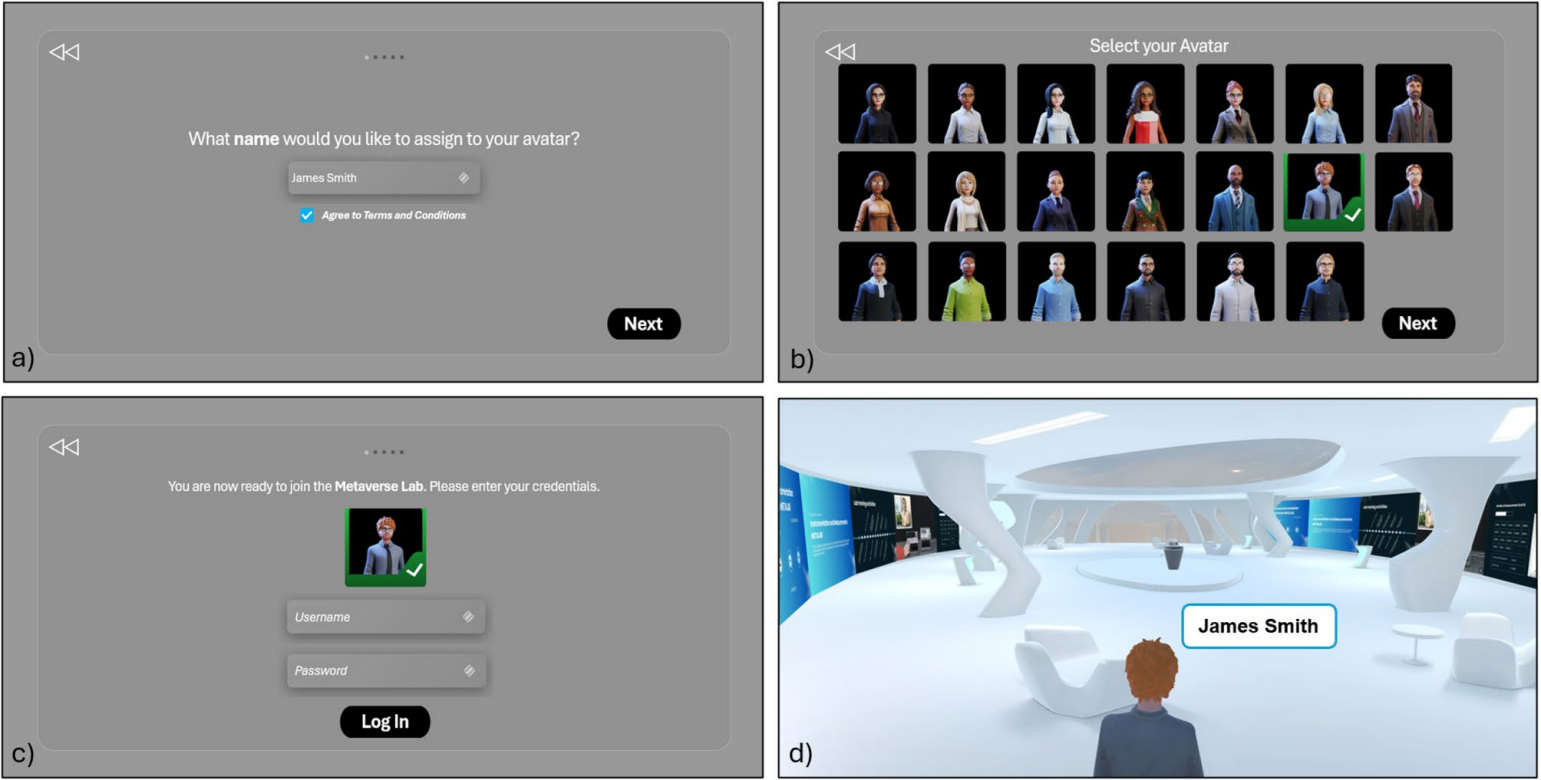
User’s view during login procedure to the IM-MetaLAB; after name (**a**) and avatar (**b**) selection, students have to provide username and password for authentication and authorization (**c**) in order to access the IM-MetaLAB (**d**).

To access the laboratory in the Metaverse, the teacher and students must (*i*) wear VR headsets, (*ii*) connect to the server, (*iii*) enter their username, (*iv*) select their desired avatar, and (*v*) log in by entering their credentials. A log file associated with each login allows for assessing learning and obtaining feedback on each student’s understanding of the course topics. The real user with VR accessories and their view during login are shown in Figs. 9 and 10, respectively. Once entered in the Metaverse, the student can access all available learning resources (videos, presentations, manuals, and books).

The opportunity of interfacing with the instruments is undoubtedly the most innovative educational aspect of the IM-MetaLAB. The measurement setup in the Metaverse is the digital twin of the measuring station in the real lab. The students interact with the instruments, pressing buttons on the front panel, exactly as they would do in the real lab, and, actually, they are really working with the real instruments. In fact, each operation of the user produces the remote connection with the real instrument in the lab and the transmission of the message for the real instrument to perform the operations requested by the user in the Metaverse.

A core innovation of the IM-MetaLAB is the direct, real-time connection between the virtual digital twins and their corresponding physical instruments. This connection enables students to operate real devices through a 3D immersive interface, with the virtual replica continuously synchronized with the real instrument’s configuration and data.

Unlike fully simulated environments, this architecture allows users to interact with actual laboratory equipment remotely—providing both realism and practical value. The backend architecture has been deliberately designed to ensure flexibility and scalability, accommodating diverse institutional setups without requiring proprietary or costly upgrades.

The solution builds upon well-established standards in educational laboratories. Instruments are connected via the General Purpose Interface Bus (GPIB, IEEE-488), a protocol that allows control of multiple instruments through a shared communication bus^29,30^. Instrument commands follow the SCPI specification. For example, a SCPI query such as “MEAS:VOLT:DC?” instructs a multimeter to measure Direct Current (DC) voltage.

Traditionally, these commands are sent from LabVIEW-based desktop software via a GPIB interface. However, to support remote access, a dedicated server must replace the local PC, accepting SCPI commands over the internet and relaying them to the instruments. This is typically implemented using Transmission Control Protocol/Internet Protocol (TCP/IP), Hypertext Transfer Protocol (HTTP), Hypertext Transfer Protocol Secure (HTTPS), or WebSocket protocols.

In IM-MetaLAB, this infrastructure is extended through an Internet-of-Things-oriented protocol: MQTT (Message Queuing Telemetry Transport), chosen for its lightweight, low-latency, and scalable architecture^31^. MQTT operates on a publish/subscribe model, with a central *Broker* managing message exchange between publishers (e.g., the Metaverse application) and subscribers (e.g., instrument servers)^32^. The choice of MQTT for real-time communication between the virtual environment and physical instrumentation was also guided by its low-latency characteristics. In our previous work on an augmented reality remote laboratory^14^, we conducted a detailed latency analysis of the MQTT-based control architecture. The experimental results demonstrated that command-and-response cycles between the client and laboratory instruments consistently remained below 100 milliseconds, with average latencies in the range of 20–50 milliseconds under typical academic network conditions. Such latencies are negligible in the context of remote measurement activities and ensure the responsiveness needed for a smooth user experience in immersive environments. To ensure secure interaction with physical instruments, the MQTT broker deployed in the IM-MetaLAB framework supports user authentication and transport-layer encryption via the Transport Layer Security (TLS) protocol. Each client (either a student interface or backend server) must authenticate using valid credentials before subscribing or publishing to any topic. This setup prevents unauthorized access to measurement equipment and ensures the integrity and confidentiality of command and data streams. These features are critical for maintaining safe and controlled access in a distributed educational environment, especially when the system is open to remote connections over the internet.

Each message in MQTT is composed of a *topic*, identifying the communication channel (e.g., the instrument to be addressed) and a *payload*, containing the command, query or response data. The hierarchical topic structure used in IM-MetaLAB supports scalability and readability. As an example, when the student press the VDC button on the digital twin of the multimeter, the following MQTT message is generated:

- topic: Metalab1/Station1/Instrument2
- payload: “MEAS:VOLT:DC?”

Here, Metalab1 identifies the lab instance, Station1 the experimental bench, and Instrument2 contains the GPIB address (2) of the specific device. This naming convention allows easy routing and supports the addition of new stations or instruments without system reconfiguration.

The control logic on the client side (i.e., within the Metaverse environment) is implemented using JavaScript. User actions on virtual instruments are captured by event-driven handlers, which translate gestures (e.g., pressing a button) into the corresponding SCPI string. These strings are then published to the MQTT broker, which dispatches them to the appropriate subscriber (i.e., the real lab PC server).

The LabVIEW server, configured as an MQTT subscriber using the LabVIEW MQTT Toolkit^33,34^, processes these messages by subscribing to all topics under Metalab1/Station1/#, receiving both commands and metadata. Once received the MQTT message from the broker, the server parses the topic string to identify the target instrument, extracts the SCPI command from the payload, and sends the command via GPIB using the LabVIEW Write function.

If the command requires a response (i.e. consists of a so-called *query*), the system performs the reverse process. The response is read using the LabVIEW Read function and republished to a designated MQTT response topic (e.g., Metalab1/Station1/Instrument2/Responses for the considered example). This separation ensures clarity, enables debugging, and takes full advantage of MQTT’s asynchronous messaging capabilities.

Importantly, this architecture supports full extensibility. To add a new instrument, it is sufficient to (*i*) develop the corresponding digital twin model, (*ii*) implement its event logic in the Metaverse, (*iii*) connect it physically to the GPIB-enabled server, and (*iv*) register the appropriate MQTT topic in the broker. This modular structure ensures compatibility across diverse instruments and lab setups, making the IM-MetaLAB framework a robust and future-proof solution for immersive, instrumented education^35,36^. This infrastructure, while technically sophisticated, was intentionally designed to be transparent to the student. The underlying MQTT-based communication with real instruments enables a seamless and authentic lab experience, preserving all pedagogical objectives of hands-on measurement training in a fully virtual environment.

## Case study: measurement of RMS voltage through the multimeter

Laboratory experiments in measurement courses serve two fundamental objectives: (1) enabling students to become familiar with the operation of instrumentation; and (2) helping them choose and correctly apply appropriate measurement techniques. Furthermore, group exercises emulate professional environments where teamwork, collaboration, and adaptability are essential. However, in traditional labs, these exercises can suffer from uneven participation and limited individual feedback, making it difficult for instructors to assess student contributions.

IM-MetaLAB addresses these issues by combining immersive interaction with automatic tracking of user actions. Avatars and gamified environments promote engagement, while the use of virtual digital twins of real instruments removes risks associated with costly or hazardous equipment. The system logs interactions for each avatar, allowing instructors to assess individual effort and guide students via automated feedback mechanisms. These features also facilitate the creation of personalized learning paths.

According to learning path, students must complete 5 lab experiences, characterized by different levels of difficulty. To demonstrate IM-MetaLAB capability and advantages, the attention will be focused here on a typical exercise, the measurement of the RMS value of a sinusoidal voltage. As in the physical lab, the activity begins with a theoretical briefing, where the instructor introduces the concept of RMS voltage, explains how to configure a signal generator and multimeter, and discusses repetition, statistical analysis (mean, standard deviation), and measurement uncertainty.

In the physical setting, students connect a signal generator to both a multimeter and an oscilloscope, using the latter to visualize the waveform and verify the reading. In the IM-MetaLAB, the same process is carried out via virtual controllers and instrument digital twins. The platform automatically detects which student has connected each component, and verifies correctness relative to the experiment goals—providing instant, context-aware feedback.

**Fig. 11:**
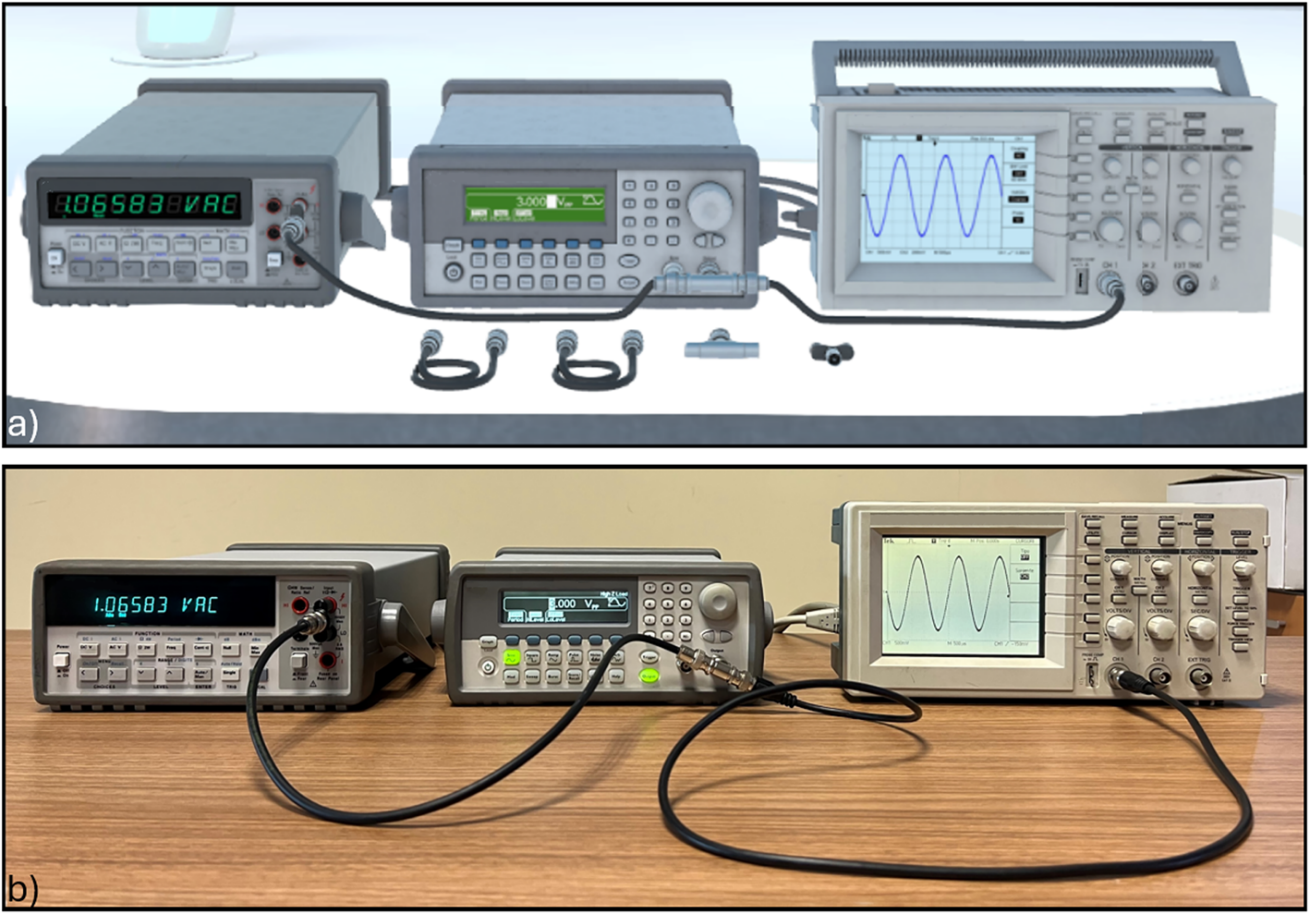
Measurement circuit (**a**) in the IM-MetaLAB and (**b**) in the real lab for the RMS voltage measurements.

As shown in Fig. 11, the virtual setup mirrors the real-world configuration. The signal generator is set to output a 3 V_pp_ sinusoidal waveform at 600 Hz. The multimeter is configured to read the RMS value, which in the shown instance is 1.06583 VAC.

A crucial feature of IM-MetaLAB is the real-time synchronization between virtual and physical instruments. As stated above, user interactions on the virtual multimeter are translated into SCPI commands and transmitted via MQTT to the physical instrument. Once the measurement is completed, the result is sent back to the virtual multimeter and displayed to the user, maintaining full coherence between the two environments.

**Fig. 12:**
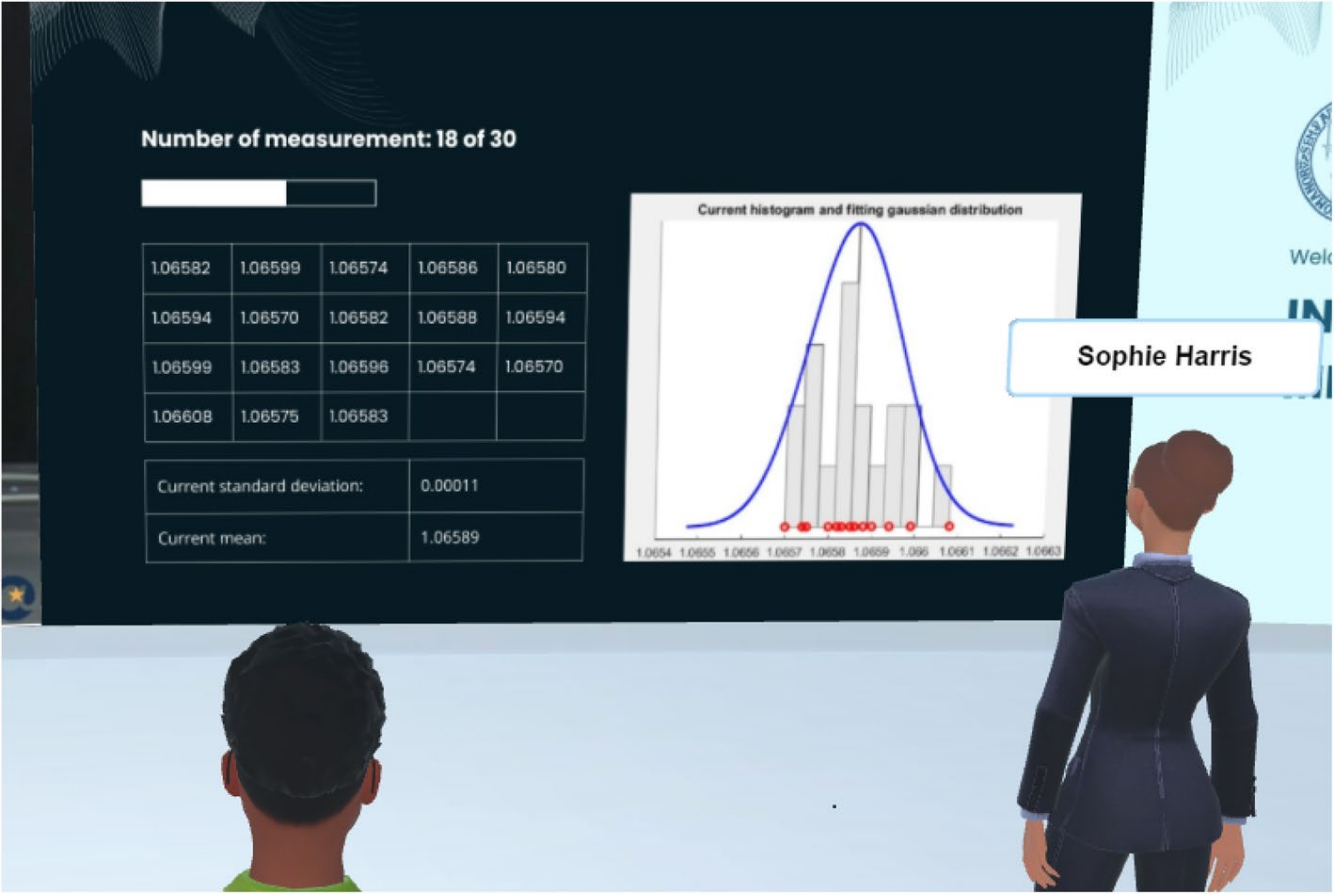
Wall screen adopted as dashboard by students; each obtained reading from the instrument is added to the table and expolited to calculate statistics and distribution fitting.

Students are asked to perform 30 repeated RMS measurements and calculate the corresponding average and standard deviation for the successive estimation of the A-type contribution to the uncertainty. In the IM-MetaLAB, this process is enhanced by integrated dashboards that display measurement data in real time (Fig. 12). These dashboards include a progress bar tracking completed measurements (e.g., 18 of 30), a dynamic table of measurement values with updated statistical metrics, and a histogram with Gaussian curve fitting for distribution analysis.

By combining experimental accuracy with immersive feedback and interactive analytics, IM-MetaLAB transforms traditional measurement exercises into rich, data-driven, and highly participatory learning experiences.

### Operating steps and sequence diagram

The following description illustrates the internal operation of the IM-MetaLAB platform during the case study previously introduced. The actions are derived from an actual immersive session, where a student configures a sinusoidal waveform and performs an RMS voltage measurement using real laboratory equipment.

Upon entering the virtual laboratory, the student interacts with the digital twin of an arbitrary waveform generator, whose GPIB address is 12. The virtual instrument replicates all the front panel elements of the real device. The student begins by selecting the desired waveform: using a VR controller, they point at the instrument front panel and press the button “Sine”. This triggers the publication of an MQTT message containing the corresponding SCPI command:

FUNC SIN $\rightarrow$ Metalab1/Station1/Instrument12/Commands

The student then sets the signal frequency; to this aim, thanks to the soft touch buttons just under the display and the available numeric keypad, the student inputs “600”, selecting “Hz” as the unit. Only after pressing the “Done” button, the value is shown on the instrument display and the SCPI command is dispatched:

FREQ 600 $\rightarrow$ Metalab1/Station1/Instrument12/Commands

The same interaction flow is used to set the amplitude (VOLT 3) and DC offset (VOLT:OFFS 0), each followed by explicit confirmation.

Once all parameters are configured, the student enables the output by toggling the “Output” button on the digital twin. A virtual Light Emitting Diode (LED) lights up, indicating activation. This action sends the SCPI command:

OUTP ON $\rightarrow$ Metalab1/Station1/Instrument12/Commands

Each of these MQTT messages is received by a LabVIEW server subscribed to the appropriate topic. The server parses the payload and issues the corresponding SCPI command over GPIB to the real function generator at address 12. The actual instrument updates its configuration in real time and begins producing the requested signal.

RMS voltage measurements are carried out by means of the multimeter, whose GPIB address is 22. The student presses the button “VAC” on the digital twin of instrument and the corresponding MQTT message is published

MEAS:VOLT:AC? $\rightarrow$ Metalab1/Station1/Instrument22/Commands

The LabVIEW server forwards this request over GPIB to the real multimeter at address 22 and the instrument responds with the RMS voltage value, which is published on the response topic:

+1.06583 $\rightarrow$ Metalab1/Station1/Instrument22/Responses

This value is rendered on the display of the virtual instrument, closing the loop between immersive action and physical feedback.

**Fig. 13:**
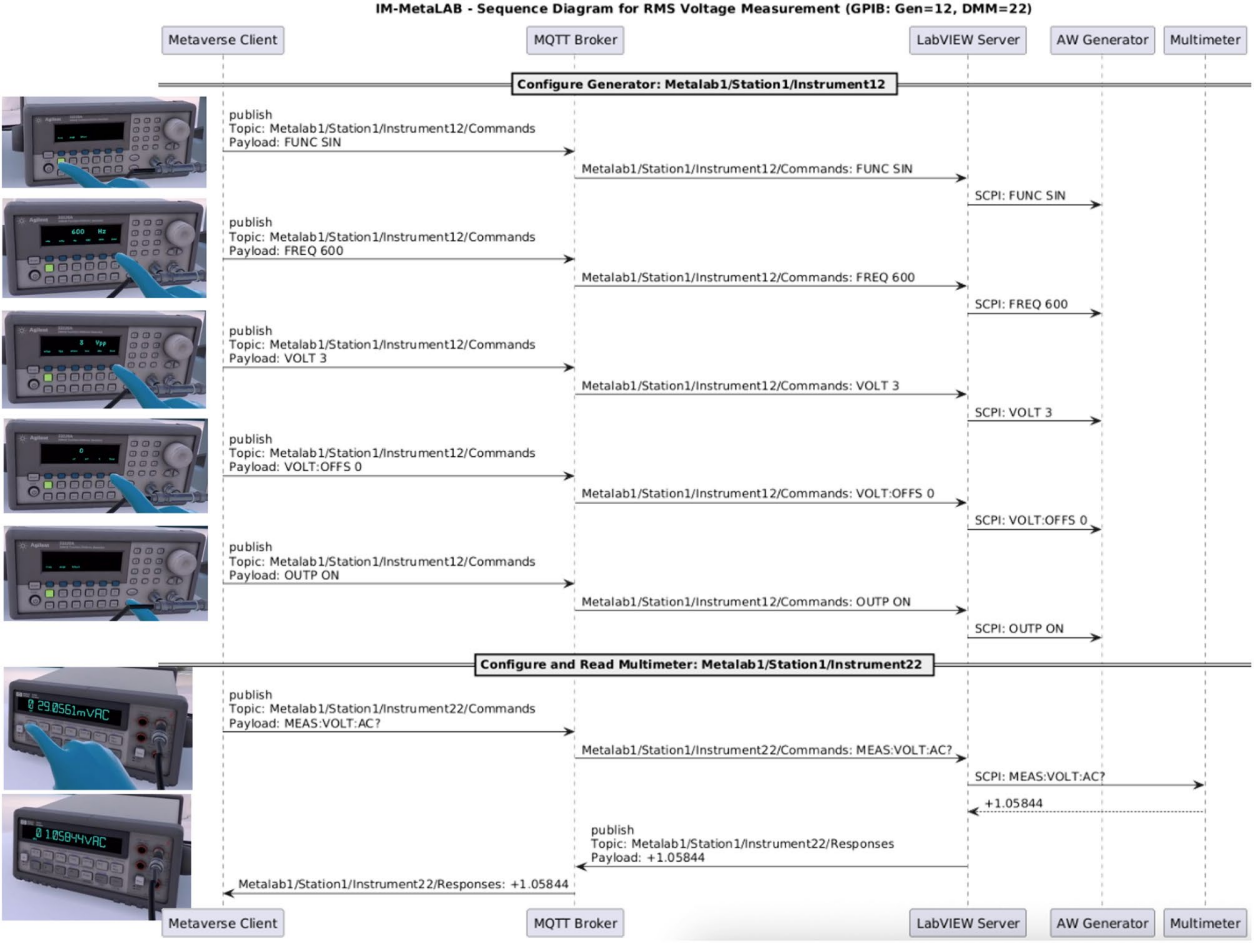
Sequence diagram illustrating the end-to-end interaction between the immersive client, MQTT broker, LabVIEW GPIB server, and real instruments during the sinusoidal RMS voltage measurement activity.

The full system-level interaction—covering user input, message routing, backend control, and physical validation—is documented in the sequence diagram shown in Fig. 13. This diagram captures each MQTT transaction, the triggering SCPI command, and the communication flow between the virtual environment and real devices. It validates the runtime correctness of the architecture and demonstrates that each immersive gesture corresponds to an actual, verifiable command exchange with laboratory instruments. Moreover, a Supplementary file (referred to as “Supplementary video 1”) consisting of a video showing all the considered operations (along with the cable connections) has been added to the paper.

## Analysis of KPIs

In order to assess the effectiveness of the implemented IM-MetaLAB, KPIs were examined and evaluated.

The results were compared among the following teaching methods for the fundamentals of measurement instruments:

1. Courses in which the laboratory session is conducted in the IM-MetaLAB; data were taken from 80 students of the “Measurement Fundamentals” course of the current academic year (2024/25).
2. Traditional courses with laboratory session in presence at the real University lab; data were taken from 110 students of the course of “Measurement Fundamentals” of the last academic year (2022/23).
3. Courses totally online, where the laboratory part is also carried out online; the data were taken from 60 students enrolled in the MOOC of “Measurement for Industrial Production and Automation” delivered on the Learning Management System (LMS) platform provided by the University of Naples Federico II^37^.
4. Online courses involving interaction with real laboratory instruments through remote control apps; data were taken from 75 students of the course of “Measurement Fundamentals” of the academic year 2020/21; during the pandemic restrictions, the authors provided the students with an augmented reality app for the remote control of the real laboratory instrumentation^14^.

The indices were evaluated by considering the following data sources: 1) Logs of accesses to the IM-MetaLAB 2) Logs of operations performed by students on the instruments 3) Course grades of students 4) Responses to surveys administered to students.

These indicators, derived from real classroom deployment over multiple academic years, demonstrate that IM-MetaLAB is not a theoretical concept but a validated, scalable solution for instrumentation education in higher education settings.

### Utilization time per student of the lab

It has to be considered that in the traditional courses, the laboratory is equipped with 8 identical measurement stations. The students were required to divide into groups of 4 and each group had the availability of one station. The case study takes into account the students of the course “Measurement Fundamentals”, who were more than 48 in number; therefore, several shifts were required. For each exercise, each group was allowed about 80 minutes in the Lab. This means that in the traditional face-to-face course, the average time per student for an exercise is approximately 80 minutes divided by 4, i.e. 20 minutes. Added to this is the stress of the lecturer having to repeat the theoretical concepts associated with a lab exercise several times, with an inevitable loss of effectiveness.

With regard to the IM-MetaLAB, the average time of laboratory use per student was measured by checking the log of student accesses and disconnections. The index showed that for one exercise, each student used the Lab for approximately 2 hours. It should be noted that the IM-MetaLAB registers the peak of accesses in the evening hours. This may be due both to students who, following the in-presence lecture, wish to deepen the concepts they have learnt with further experimental sessions, and to working students who, thanks to this solution, can practice at times that are more favorable to their work commitments.

As regards the other training methodologies, the increase in available Lab time is an advantage characterizing also courses using apps for remote Lab connection; on the contrary, the Lab time is zero for courses involving exclusively online training.

### Completion of course activities

Five experimental activities are proposed to the students during the course. The students, after understanding the exercise, have to carry it out independently and submit a report.

**Fig. 14:**
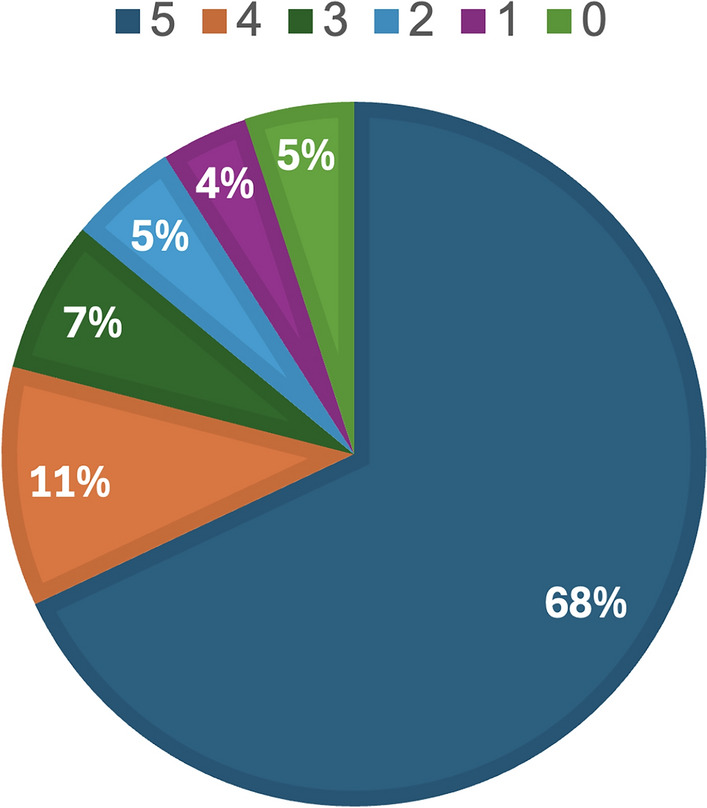
Activities completed two months after the end of the course.

Based on the reports received, the percentage of completion of the activities by all students in the course was assessed. In Fig. 14, the activities completed by the student of the course employing the IM-MetaLAB are reported. Two month after the end of the course, 68 % of the students completed all the 5 activities. The 11 % completed 4 activities, the 7 % completed 3 activities, the 5 % completed 2 activities, and the 4 % completed 1 activity. Finally, the 5 % of the students did not complete any activity; this is probably the percentage of students who, for various reasons, abandon the course in its initial stages. When comparing the number of students who completed all the proposed activities with the corresponding number observed in the traditional course (56 %), a significant increase can be seen. The greater time availability offered by the IM-MetaLAB, in fact, allows students to go deeper into the procedures explained by the lecturer and successfully complete the experimental activities. It is worth noting that this index is also significantly higher than the corresponding index observed for courses that allow the use of the remote laboratory (62 %). The authors interviewed students and monitored the accesses to try to understand the reasons for this difference and received confirmation of an important feature of the IM-MetaLAB: the possibility of meeting other students, having social interactions and carrying out group activities. Even from their own homes, students autonomously decided to meet themselves in the Metaverse and work together. This opportunity certainly has benefited their overall preparation.

### Achieved mean grade

The advantages of the IM-MetaLAB are also evident when looking at this index, that in this case in equal to 27.8. In the case of the traditional courses, the average grade achieved by students was 26.2. In the case of the course with the use of the remote laboratory, the average was 26.9. Finally, in the case of the fully online course, the deterioration in the students’ learning is perceptibly observable; the average mark awarded was 24.5. In this regard, it should be specified that the first part of the examination consists of replicating, in front of the lecturer, one of the experimental laboratory tests performed during the course. Therefore, the grade obtained in the exam certainly reflects the level of preparation achieved by the student with regard to the use of laboratory instrumentation.

Again, unlimited access to real laboratory instrumentation and learning resources, combined with the discussions with other students, produces an obvious benefit in total preparation.

### Students’ opinion

A survey was administered to the students who used the IM-MetaLAB in the course. The questions touch on various aspects such as usability, usefulness and the sense of reality provided by the Lab. In particular, there were five possible answers for each question: strongly agree, agree, neutral, disagree, strongly disagree.

The perceived sense of reality is very high. The 95 % of students thinks that the digital twins of the instruments in the Metaverse are extremely faithful to the real instruments found in the laboratory during the exams.

Furthermore, the 88.8 % of the students perceived that they were controlling a real instrument from the Metaverse; this result is not easy to achieve when people operate in virtual environments.

As expected, a large amount of students (87.5 %) believe that using the Metaverse Lab helped them better understand the theoretical concepts of the course. The 86.3 % of students found the IM-MetaLAB simple to use, without requiring the teacher’s support. The use is so straightforward, that the 76.3 % of students even think that the IM-MetaLAB could replace sessions in the real laboratory, without lack of competencies.

**Fig. 15:**
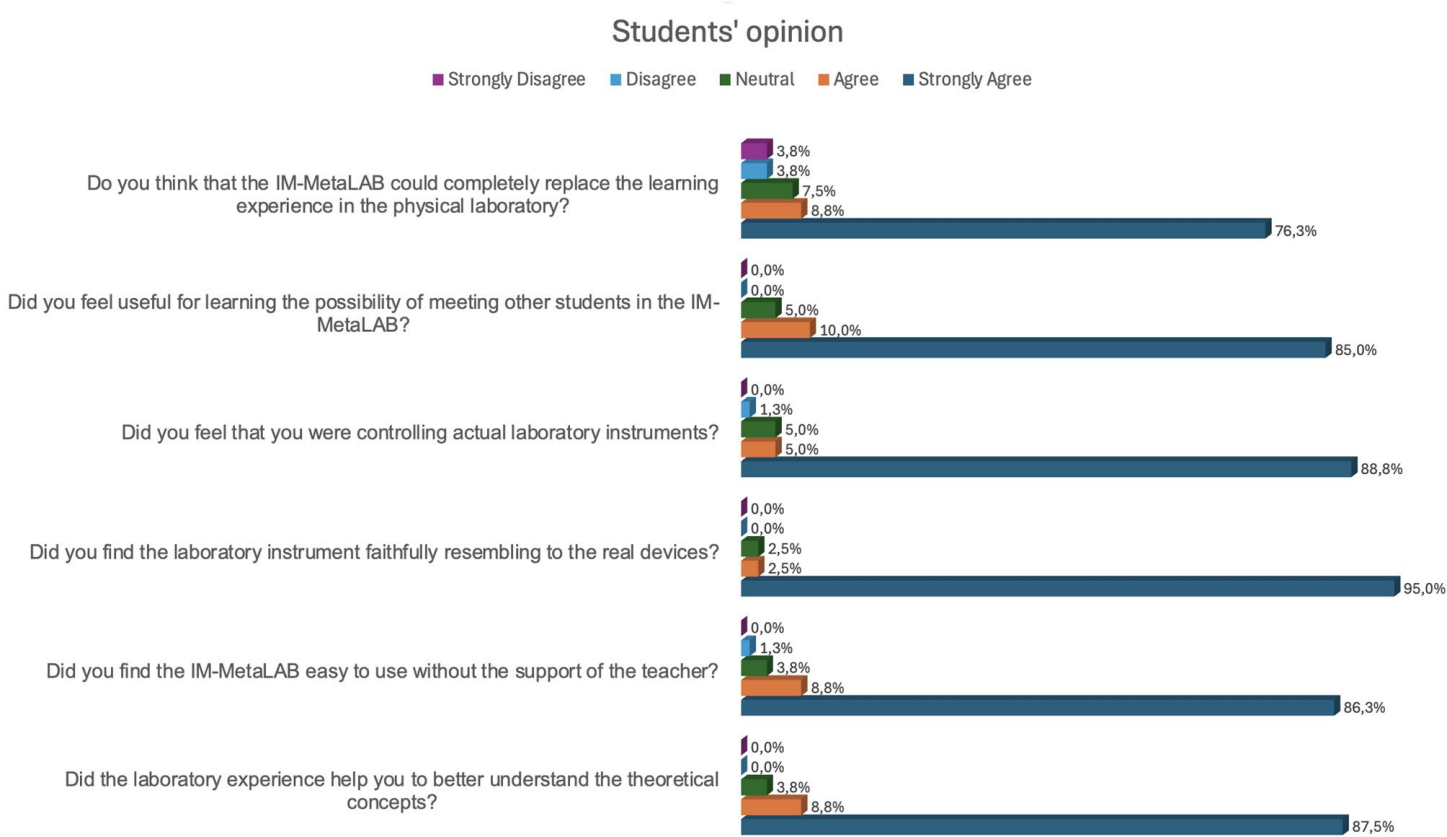
Students’ opinion about the IM-MetaLAB.

Finally, students appreciated the opportunity to meet each other in the Metaverse; the features of the metaverse allow them to collaborate and receive exhaustive explanations from the teacher, even if they are physically located in a place far from the university. In Fig. 15 students’ opinion has been reported.

The survey responses were also compared with the same data for the other course types.

**Fig. 16:**
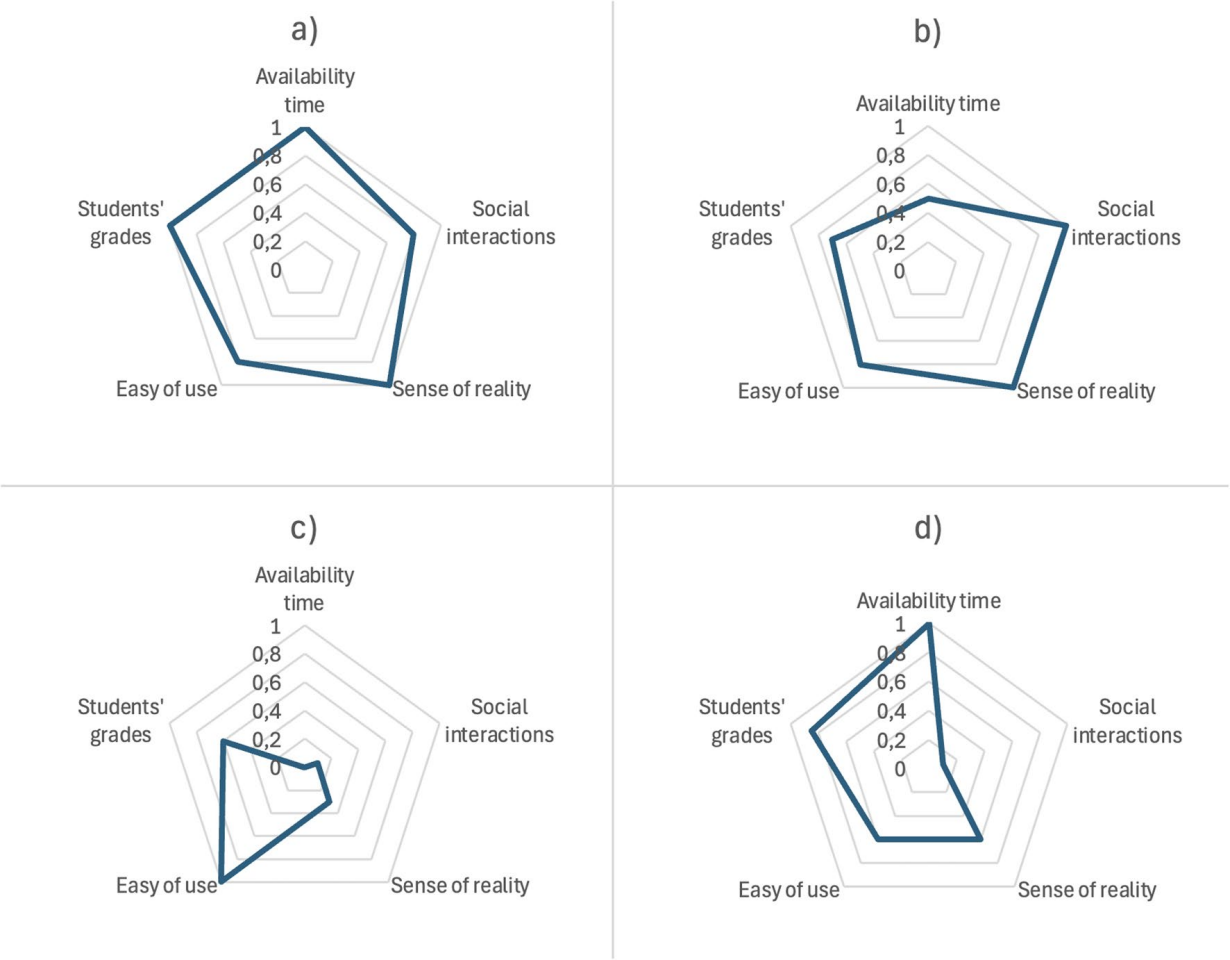
Global characteristics of the four considered training methods. (**a**) IM-MetaLAB; (**b**) Traditional Lab; (**c**) Online course; (**d**) Remote Lab. As it can be appreciated, IM-MetaLAB is capable of merging and leveraging the advantages of remote and in-presence laboratory, thus proving as an efficient tool for didactical purposes.

In order to summarize the strengths and disadvantages of each training method, a diagram highlighting the main characteristics is shown in Fig. 16.

The IM-MetaLAB is the teaching method that offers the most advantages in the different areas considered. In fact, it offers the maximum availability time per student, as apps for remote control do. Still, the sense of reality, the engagement and social interactions are equal to those guaranteed by traditional lessons in the laboratory.

## Summary

This paper presented the IM-MetaLAB, an immersive and interactive laboratory environment designed for teaching instrumentation and measurement. The system leverages the concept of digital twins, which replicate physical instruments within a photorealistic virtual reality environment and allow students to interact with real devices through MQTT-based communication and SCPI command translation. The platform integrates virtual reality with real-time control of laboratory instruments, enabling students to carry out remote experiments in a fully immersive and multi-user context.

Key performance data highlight the superior effectiveness of this approach compared to traditional, fully online, and remote lab methods. Specifically, 70% of students in the IM-MetaLAB completed all required laboratory activities within two months, significantly outperforming the 56% completion rate observed in the traditional lab setting. This improved completion rate underscores the impact of flexible, on-demand lab access, which allows students to deepen their understanding of complex concepts through self-paced exploration. Additionally, student exam scores reflect a notable enhancement in learning outcomes, with an average grade of 27.4 out of 30, surpassing the 26.1 average in traditional labs and 24.8 in fully online courses. Student feedback further supports these findings, with 97.5% of participants attesting to the high fidelity of digital twins to real-world instruments and over 90% reporting that the IM-MetaLAB enhanced their grasp of theoretical concepts. The IM-MetaLAB’s structure also encourages collaborative and social learning, helping students develop essential soft skills like teamwork and communication, which are less accessible in isolated remote learning setups. Through real-time interactions and shared tasks in the virtual lab, students reported an enriched learning experience, emphasizing engagement and a sense of community.

Numerous studies have examined both the benefits and challenges associated with integrating technology into education^38–43^. However, the focus of this paper is not on evaluating the learning improvement specifically attributed to the metaverse environment as a didactical tool. Instead, the observed enhancement in student performance is primarily linked to the 24/7 availability of laboratory resources, which enabled students to practice more extensively with measurement instrumentation. This increased familiarity and confidence with the measuring stations is directly reflected in the higher average grades achieved under this condition compared to the other considered scenarios. For this evaluation, data were drawn from the departmental database, which required no additional consent, as authorization was inherently granted upon student enrollment. The comparative analysis presented in Sect. "Analysis of KPIs" highlights the effectiveness of IM-MetaLAB across several learning dimensions. The increased lab time per student (Fig. 13), higher task completion rates (Fig. 14), improved average exam grades (Fig. 15), and positive student feedback (Fig. 16) collectively validate the system’s educational value. These quantitative findings support the qualitative observation that immersive, real-instrument-linked virtual environments foster deeper understanding, better engagement, and broader accessibility. These aspects are critical, especially in technical education where practical training is essential. The current validation effort confirms that IM-MetaLAB is not only technically viable but also pedagogically impactful.

## Future work

To further enhance the flexibility and scalability of the IM-MetaLAB, ongoing development focuses on replacing the current LabVIEW-based server infrastructure with a custom, low-cost hardware interface. This homemade solution is designed to directly translate MQTT messages into SCPI commands transmitted via the GPIB protocol, effectively decoupling the system from commercial software platforms. By reducing hardware and licensing costs while preserving full compatibility with existing laboratory equipment, this approach aims to facilitate broader adoption of the IM-MetaLAB framework across diverse educational institutions, including those with limited technical or financial resources.

Future activities will also explore improved synchronization mechanisms, the integration of haptic feedback devices to enhance realism, and the deployment of edge computing resources to reduce latency in real-time interaction with remote instruments. These developments will further reinforce the IM-MetaLAB as a scalable, modular, and inclusive platform for immersive laboratory education.

## Supplementary Information

Below is the link to the electronic supplementary material.

Supplementary material 1.

Supplementary material 2.

## Data Availability

The datasets generated and/or analyzed during the study are available from the corresponding author upon reasonable request and subject to conditions that ensure the confidentiality of the participants is safeguarded.
