# Supplementary material for: IM-MetaLAB: the first digital laboratory for teaching the fundamental concepts of instrumentation and measurement in metaverse
Source: Sci Rep. 2025 Nov 26;15:45401. doi: 10.1038/s41598-025-29553-7 (PMC12749875; doi:10.1038/s41598-025-29553-7)
Supplement: Supplementary file 1 — Supplementary material 1. [file 41598_2025_29553_MOESM1_ESM.pdf]

Supplementary Video Legend

Supplementary Video 1 - IM-MetaLAB operational workflow.

The video shows a complete experimental session carried out in the IM-MetaLAB immersive environment. A student configures a sinusoidal waveform on the digital twin of an arbitrary waveform generator, setting the frequency to 600 Hz, amplitude to 3 Vpp, and zero DC offset. Each action on the virtual instrument corresponds to an MQTT message and a SCPI command sent in real time to the physical instrument (GPIB address 12). The output is then enabled, and the RMS voltage is measured using the digital twin of a digital multimeter (GPIB address 22). The measured value, returned by the real instrument, is displayed on the virtual device, confirming the full bidirectional synchronization between the immersive interface and the physical laboratory setup.
